# Association between magnesium, copper, and potassium intakes with risk of rheumatoid arthritis: a cross-sectional study from National Health and Nutrition Examination Survey (NHANES)

**DOI:** 10.1186/s12889-023-16906-y

**Published:** 2023-10-24

**Authors:** Jianguo Fang, Tingwei Cao, Cai Liu, Duojun Wang, Hui Zhang, Jinyu Tong, Zaijun Lin

**Affiliations:** 1 Department of Spine Surgery, Shidong Hospital Affiliated to University of Shanghai for Science and Technology, No.999, Shiguang Road, Shanghai, 200438 China; 2 Department of Orthopedic Surgery, The Affiliated Hospital of Panzhhua University, Panzhihua, 617000 Sichuan Province China

**Keywords:** Rheumatoid arthritis, NHANES, Magnesium, Copper, Potassium

## Abstract

**Background:**

The relationship between Mg (magnesium), Cu (copper), and K (potassium) intakes and the risk of rheumatoid arthritis (RA) remains limited. The aim of present study was to examine the associations between Mg, Cu and K intakes with RA.

**Methods:**

Using data from the National Health and Nutrition Examination Survey (NHANES) 2003–2018, we examined the association between Mg, Cu and K intakes and the risk of RA among US adults. After adjustment for age, sex, race, BMI, educational level, smoking history, alcohol consumption, family Poverty Income Ratio (PIR), diabetes and total daily energy intake, logistic regression models and smooth curve fitting were applied to examine the associations of Mg, Cu and K intakes with RA.

**Results:**

A total of 18,338 participants were included (1,008 participants with RA). The multivariate adjusted ORs (95% CI) of RA were [0.66 (0.51, 0.84)], [0.76 (0.60, 0.97)], and [0.75 (0.58, 0.97)] in the highest versus lowest quartile of magnesium intakes, respectively. A nonlinear association between Cu intakes and RA was found. When Cu intake (ln) was between 0.6–2.2 mg, the risk of RA reduced by 26% for every 1 mg increase of intake in Cu [0.74 (0.58, 0.96)].

**Conclusions:**

Higher Mg, Cu and K intakes may be inversely associated with the risk of RA among US adults, and an inverse L-shaped association between dietary Cu and RA was found.

## Introduction

Rheumatoid arthritis (RA) is a chronic, systemic autoimmune disease that affects the joints and organs, including the skin, eyes, lungs, heart, and blood vessels [1]. It is characterized by painful, swollen, and stiff joints, resulting in reduced mobility and decreased quality of life [2]. RA can also cause systemic inflammation, leading to fatigue, fever, and anemia [3, 4]. Estimations suggested that absenteeism and work disability related to RA accounted for a minimum of 39%, leading to a substantial economic impact [5]. Although RA is a lifelong condition, it can be managed and prevented with lifestyle changes such as healthy dietary choices and physical activities, allowing people to live active and fulfilling lives [6, 7].

Essential microelements such as Mg (magnesium), Cu (copper), and K (potassium) and Fe (iron), hold significant relevance within the human body, as they contribute to a multitude of physiological processes, such as enzymatic activity, cellular signaling, and oxygen transportation [8, 9]. There exists scientific evidence indicating that certain metals may help reduce inflammation associated with RA, as well as provide other potential benefits [10–12]. For instance, zinc have antioxidant properties, which may serve to protect joint tissue from damage caused by reactive oxygen species [13]. Mg, another essential trace element, has been linked to a reduced risk of RA in observational studies, possibly due to its function in modulating the immune response and inhibiting proinflammatory cytokine production [14–16]. Moreover, Cu is an essential component of several enzymes involved in the synthesis and stabilization of connective tissue, and dysregulation of Cu metabolism may be involved in the pathogenesis of RA [17, 18]. Despite these previous findings, to our knowledge, research on the association between the consumption of Mg, Cu and K and the risk of RA remains limited.

Therefore, we conduct the present study to investigate the potential association between the consumption of Mg, Cu and K and the risk of RA using data from the National Health and Nutrition Examination Survey (NHANES) from 2003–2018.

## Method

### Study population

The NHANES is a cross-sectional survey designed to collect information on the health, nutrition, and sociology from all levels of population in the United States [19]. Survey participants received in-home interviews, followed by a visit to a mobile examination center (MEC) for various examinations and laboratory measurements. Observations were based on eight independent NHANES cycles including 2003–2004, 2005–2006, 2007–2008, 2009–2010, 2010–2011, 2013–2014, 2015–2016 and 2017–2018. All the participants provided written informed consent before the examinations, which was conducted by the National Center for Health Statistics (NCHS) ethics review board.

To avoid the bias caused by other types of arthritis, we excluded participants with reporting arthritis other than RA. A total of 18,338 individuals (10,042 males and 8,296 females) aged over 20 years have been included in this study, and 1,008 (5.5%) participants had been diagnosed as RA.

### Assessment of RA

The diagnosis of RA was determined using the self-reported personal interview data on health condition questionnaires. At first, participants were asked “Has a doctor or other health professional ever told you that you had arthritis”. The response includes “Yes” or “No”. If the answer was “Yes”, the next question “Which type of arthritis” would be asked. The participants whose answer indicated RA were considered to have RA.

### Dietary intakes

The NHANES provides information on two 24-h dietary recall interviews of the participants. The first interview is conducted in-person at the Mobile Examination Center (MEC), while the second interview is conducted via telephone three to ten days later. In our study, we calculated dietary intake estimates using the average of data from two dietary recalls. If one of the 24-h interviews was missing, we utilized the data from the single day recall to estimate dietary intake.

### Covariates

Based on clinical knowledges and previous studies, we chose some covariates related to the disease of RA in our study [20, 21]. Covariates included age (years), sex (males or females), race (non-Hispanic White, non-Hispanic Black, Mexican American, or other races), education level (under high school, high school or equivalent, above high school), alcohol consumption (defined as having at least 4 alcohol drinks every day), smoking history (classified based on whether an individual has a lifetime history of smoking at least 100 cigarettes), body mass index (BMI) (< 25, 25–30, and > 30), ratio of family income to poverty (≤ 1, 1 ~ 3, and > 3), energy intake (kcal/day) and diabetes (yes or no). Participants were considered to have diabetes: 1) self-reported doctor’s diagnosis of diabetes. 2) currently take medicine for controlling blood glucose; 3) A Hemoglobin A1c (HbA1c) level ≥ 6.5%.

### Statistical analysis

The statistical analysis was performed using R software (version 3.6.3) and the 'survey' package for complex survey data analysis. A two-tailed P value less than 0.05 was considered statistically significant. Because of the complex survey design of NHANES, we calculated the new weight of our survey data according to the analytical guideline edited by NCHS.

Due to the considerable skewness towards the upper end in the distribution of Mg, Cu and K intake values, a natural logarithm (Ln) transformation was applied for analysis. Continuous variables were presented as means with standard deviations (SD), while categorical variables were expressed as frequencies and percentages. Weighted two-tailed t-tests were employed for continuous variables, and weighted Rao-Scott chi-square tests were utilized for categorical variables. Intakes of dietary Mg, Cu and K were stratified based on quartiles (Q1: < 25th percentile, Q2: ≥ 25 to 50th percentile, Q3: ≥ 50 to 75th percentile, Q4: ≥ 75th percentile). Multivariate logistic regression models were used to examine the association between the intake of Mg, Cu and K and the risk of RA, with the first quartile serving as the reference group. Subgroup analyses stratified by sex were also performed. Model 1 was adjusted for potential confounders, including age, sex, and race. Model 2 included further adjustments for BMI, poverty-to-income ratio, educational level, smoking history, alcohol consumption, daily energy intake, and diabetes.

In addition, we converted analysed exposure quantiles into continuous variables, which were then presented as the median value of each quantile. Then we conducted tests for linear trends using logistic regression. Finally, a weighted generalized additive model and a smooth curve fitting were conducted to address for non-linearity.

## Results

### Characteristics of participants

**Fig. 1:**
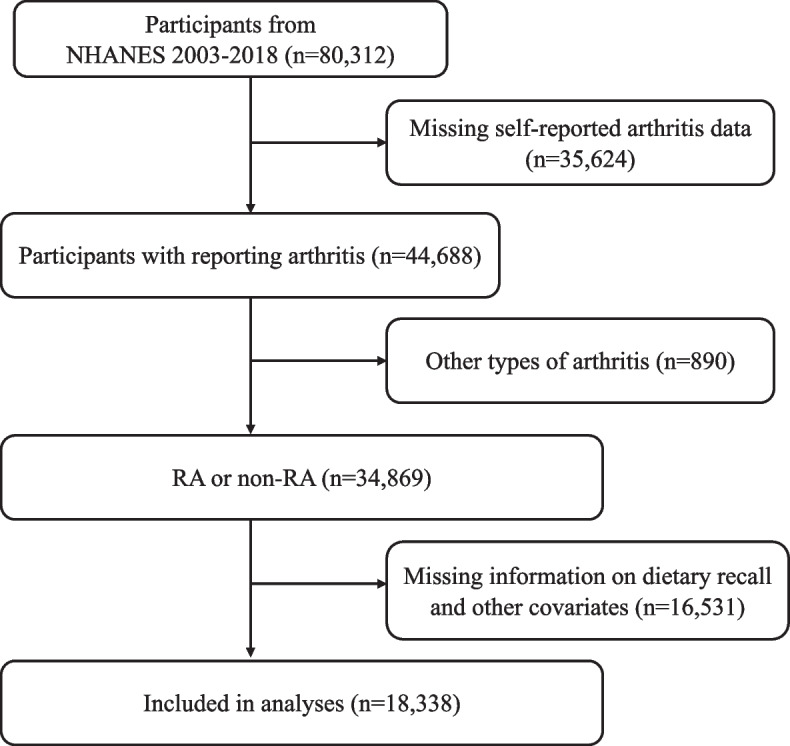
Flow chart of participants selection. Abbreviations: NHANES, National Health and Nutrition Examination Survey; RA, rheumatoid arthritis

Figure 1 showed the flow chart of participants. In comparison to the non-RA group, individuals in the RA group were observed to have higher probabilities of being overweight, older, non-Hispanic blacks, smokers, drinkers and as well as displaying a greater prevalence of diabetes and lower levels of educational attainment and income (Table 1).

**Table 1:** Baseline Characteristics of participants by RA among U.S. adults, NHANES 2003–2018

Characteristic	Non-RA	RA	*P* value
N	17330	1008	
Age(years)	41.59 ± 14.92	54.66 ± 13.65	< 0.001
Gender			
Male	9314 (53.74%)	466 (46.28%)	0.002
Female	8016 (46.26%)	542 (53.72%)	
Race/ethnicity, n (%)			
Non-Hispanic White	12044 (69.50%)	687 (68.19%)	< 0.001
Non-Hispanic Black	1747 (10.08%)	157 (15.61%)	
Mexican American	1512 (8.72%)	67 (6.61%)	
Other race/multiracial	2027 (11.70%)	97 (9.59%)	
Education level, n (%)			
Less than high school	2010 (11.60%)	165 (16.39%)	< 0.001
High school	3750 (21.64%)	253 (25.07%)	
More than high school	11,570 (66.76%)	590 (58.54%)	
Diabetes, n (%)			
Yes	1455 (8.39%)	182 (18.07%)	< 0.001
No	15,875 (91.61%)	826 (81.93%)	
Family PIR	3.21 ± 1.62	2.73 ± 1.64	< 0.001
BMI	28.22 ± 6.43	30.31 ± 7.41	< 0.001
Energy (kcal/day)	2234.06 ± 886.38	2037.12 ± 847.83	< 0.001
Alcoholic ≥ 4 drinks/day (%)			
Yes	13,165 (75.97%)	824 (81.70%)	0.002
No	4165 (24.03%)	184 (18.30%)	
Smoked > = 100 cigarettes in life (%)			
Yes	7882 (45.49%)	632 (62.30%)	< 0.001
No	9447 (54.51%)	376 (37.70%)	
Carbohydrate (g/day)	261.43 ± 114.06	236.72 ± 102.84	< 0.001
Fat (g/day)	85.55 ± 40.59	79.04 ± 38.27	< 0.001
Protein (g/day)	86.97 ± 37.33	78.48 ± 37.18	< 0.001
Total Mg (mg/day)	312.28 ± 137.54	285.79 ± 133.58	< 0.001
Total Cu (mg/day)	1.33 ± 0.82	1.22 ± 0.63	< 0.001
Total K (mg/day)	2757.02 ± 1136.06	2643.17 ± 1150.81	< 0.001

Continuous variables were presented as mean ± SD; Categorical variables were presented as n (%)

*NHANES* National Health and Nutrition Examination Survey, *RA* rheumatoid arthritis, *BMI* body mass index, *PIR* the ratio of family income to poverty, *SD* standard deviation, *n* numbers of subjects, *%* weighted percentage, *Mg* magnesium, *Cu* copper, *K* potassium

Table 2 summarizes the odd ratios (ORs) and their corresponding 95% confidence intervals (CIs) for RA based on quartiles of dietary metal intake. Table 2 summarizes the odd ratios (ORs) and their corresponding 95% confidence intervals (CIs) for RA based on quartiles of dietary Mg, Cu and K intakes. The daily Mg, Cu, K and energy intakes in participants with RA were significantly lower than those without RA. After adjustment for age, sex, race, BMI, educational level, smoking history, alcohol consumption, family Poverty Income Ratio (PIR), diabetes and total daily energy intake in multivariate analyses, dietary Mg, Cu and K intakes were negatively associated with RA. The multivariate adjusted ORs (95% CI) of RA were [0.66 (0.51, 0.84)], [0.76 (0.60, 0.97)], and [0.75 (0.58, 0.97)] in the highest versus lowest quartile of Mg, Cu and K intakes, respectively.

**Table 2:** OR (95% confidence intervals) of RA across quartiles of Mg, Cu, K intakes, NHANES 2003–2018

	Cases/participants	Crude mode	Model1	Model2
		OR (95%CI)	OR (95%CI)	OR (95%CI)
Daily dietary Mg intake				
Quartiles				
Q1 (low)	4605/18338	1 (Ref)	1 (Ref)	1 (Ref)
Q2	4567/18338	0.72 (0.61, 0.85) **	0.73 (0.62, 0.88) **	0.74 (0.62, 0.89) **
Q3	4582/18338	0.64 (0.54, 0.76) **	0.71 (0.59, 0.85) **	0.72 (0.59, 0.88) **
Q4 (high)	4584/18338	0.52 (0.43, 0.62) **	0.67 (0.55, 0.82) **	0.66 (0.51, 0.84) **
P for trend		< 0.001	< 0.001	< 0.001
Daily dietary Cu intake				
Quartiles				
Q1 (low)	4586/18338	1 (Ref)	1 (Ref)	1 (Ref)
Q2	4585/18338	0.79 (0.67, 0.54) **	0.80 (0.67, 0.95) *	0.82 (0.68, 0.98) *
Q3	4588/18338	0.67 (0.57, 0.80) **	0.76 (0.63, 0.91) **	0.80 (0.65, 0.98) *
Q4 (high)	4579/18338	0.57 (0.47, 0.68) **	0.73 (0.60, 0.88) **	0.76 (0.60, 0.97) *
P for trend		< 0.001	< 0.001	0.023
Daily dietary K intake				
Quartiles				
Q1 (low)	4587/18338	1 (Ref)	1 (Ref)	1 (Ref)
Q2	4582/18338	0.81 (0.68, 0.96) *	0.79 (0.66, 0.95) *	0.82 (0.68, 0.99) *
Q3	4586/18338	0.77 (0.65, 0.92) **	0.78 (0.64, 0.93) **	0.82 (0.66, 1.00) *
Q4 (high)	4583/18338	0.64 (0.54, 0.77) **	0.74 (0.61, 0.80) **	0.75 (0.58, 0.97) *
P for trend		< 0.001	0.001	0.023

Model 1 adjusted for age, sex, and race

Model 2 adjusted for age, sex, race, poverty to income level, educational level, smoking history, alcohol consumption, daily energy intake, and diabetes

Table 3 OR (95% confidence intervals) of RA across quartiles of magnesium, copper and potassium intakes stratified by sex, NHANES 2003–2018

Table 4 Non-linearity addressing by weighted two-piecewise linear model of copper intakes

*NHANES* National Health and Nutrition Examination Survey, *Q* quartile, *OR* odds ratio, *Mg* magnesium, *Cu* copper, *K* potassium

^*^ *p* < 0.05, ** *p* < 0.01

**Fig. 2:**
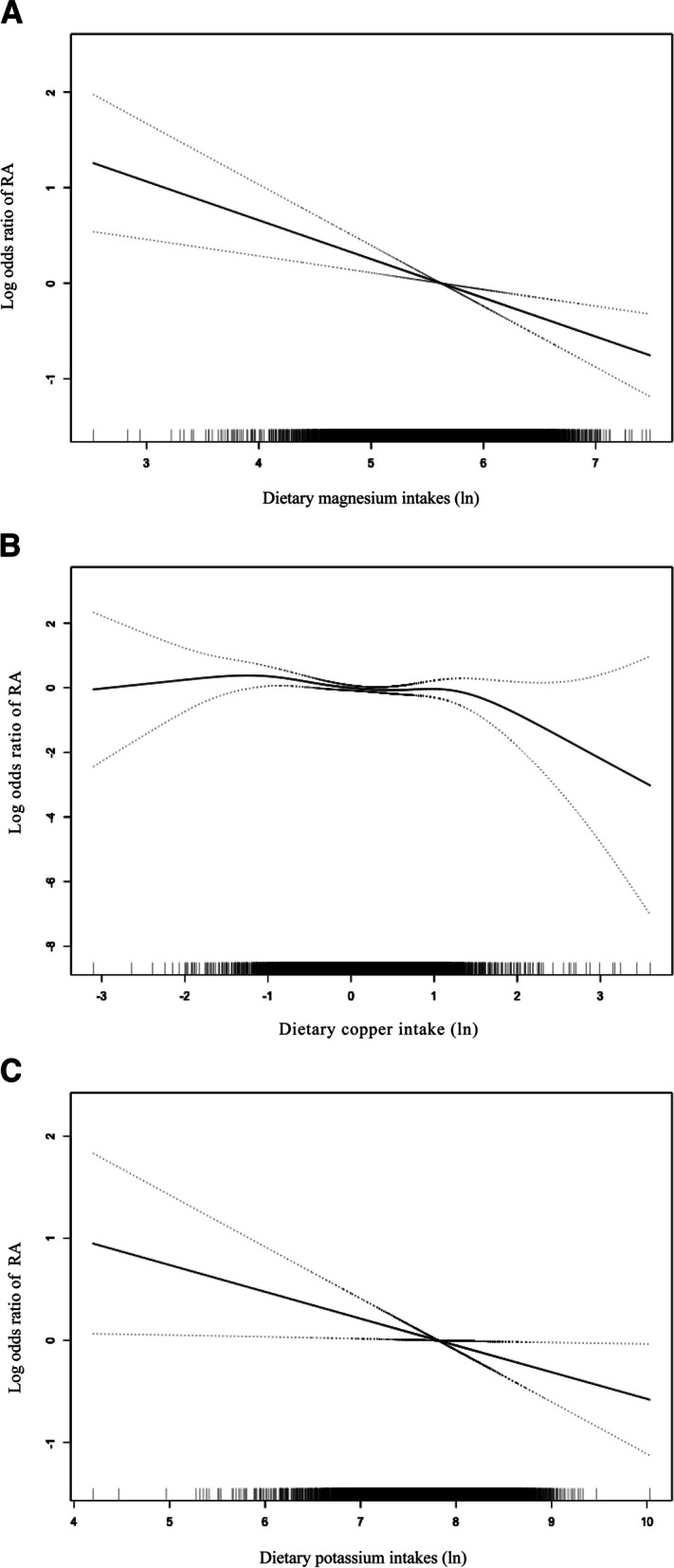
Dose–response relationship between magnesium (**a**), copper (**b**), and potassium (**c**) intakes. Adjusted for age, sex, race, poverty to income level, educational level, smoking history, drinking history, daily energy intake, and diabetes. The dashed lines represent the 95% confidence intervals

Figure 2 presents the dose-relationship between dietary Mg, Cu and K exposure and RA. The risk of RA reduced by 36% for every 1 mg increase of intake in Mg [0.64 (0.52–0.80)] (Fig. 2a), 27% for every 1 mg increase in K [0.73 (0.59–0.91)] (Fig. 2c). An inverse L-shaped association between Cu intakes and RA was found (Fig. 2b). Then, we calculated the inflection point, as shown in Table 3. The results showed that when the dietary Cu intake (ln) was below -1.2 (ie, < 0.3 mg), we didn’t observe a significant change in the relative odds of RA [0.73 (0.43, 1.23)]. However, when Cu intake (ln) was between -1.2 and 0.8 (ie, 0.3–2.2 mg), the risk of RA reduced by 26% for every 1 mg increase of intake in Cu [0.74 (0.58, 0.96)]. When Cu intake (ln) was more than 0.8 (> 2.2 mg), no significant change of the relative odds of RA was found in the relative odds of RA [0.39 (0.14, 1.08)].

**Table 3:** Non-linearity addressing by weighted two-piecewise linear model of Cu intakes

	Dietary copper intake (ln transform)	
	OR (95% CI)	*P* value
Fitting by standard linear model	0.76 (0.63–0.91)	0.003
Fitting by two-piecewise linear model		
< -1.2	1.2 (0.25,5.68)	0.816
-1.2–0.8	0.74 (0.58,0.96)	0.003
> 0.8	0.39 (0.14,1.08)	0.246
Log likelihood ratio	0.040	

Adjusted for age, sex, race, poverty to income level, educational level, smoking history, alcohol consumption, daily energy intake, and diabetes

*Cu*, copper, *OR* odds ratio

In subgroup analysis, we further explored the role of sex on the association between dietary metals and RA. As shown in Table 4, we found the ORs (95%CIs) of highest quartile for Mg in males [0.67 (0.47, 0.96)] and females [0.70 (0.49, 0.97)] were both significant. However, in the respect of Cu, the inverse association only existed in females [0.86 (0.47, 0.93)]. Regarding dietary K intakes, there was no observed significant association between Cu and RA in either males or females.

**Table 4:** OR (95% confidence intervals) of RA across quartiles of magnesium, copper and potassium intakes stratified by gender, NHANES 2003–2018

	Male			Female		
	Crude mode	Model1	Model2	Crude mode	Model1	Model2
Daily dietary Mg intake						
Quartiles						
Q1 (low)	1 (Ref)	1 (Ref)	1 (Ref)	1 (Ref)	1 (Ref)	1 (Ref)
Q2	0.77 (0.61, 0.98) *	0.87 (0.67.1.11)	0.82 (0.63, 1.06)	0.70 (0.55, 0.89) **	0.68 (0.53, 0.87) **	0.74 (0.57, 0.96) *
Q3	0.63 (0.49, 0.81) **	0.75 (0.57, 0.96) *	0.69 (0.52, 0.93) *	0.60 (0.46, 0.76) **	0.59 (0.45, 0.76) **	0.67 (0.50, 0.89) **
Q4 (high)	0.57 (0.44, 0.73) **	0.82 (0.62, 1.07)	0.67 (0.47, 0.96) *	0.55 (0.42, 0.70) **	0.58 (0.44, 0.75) **	0.70 (0.49, 0.97) *
P for trend	< 0.001	0.057	0.012	< 0.001	< 0.001	0.016
Daily dietary Cu intake						
Quartiles						
Q1 (low)	1 (Ref)	1 (Ref)	1 (Ref)	1 (Ref)	1 (Ref)	1 (Ref)
Q2	0.84 (0.66, 1.06)	0.92 (0.72, 1.18)	0.92 (0.71, 1.18)	0.73 (0.57, 0.93) **	0.69 (0.54, 0.88) **	0.75 (0.57, 0.97) *
Q3	0.73 (0.57, 0.94) *	0.88 (0.68, 1.14)	0.88 (0.68, 1.14)	0.73 (0.57, 0.92) **	0.74 (0.58, 0.95) *	0.83 (0.63, 1.11)
Q4 (high)	0.65 (0.50, 0.84) **	0.90 (0.68, 1.17)	0.90 (0.68, 1.17)	0.50 (0.38, 0.65) **	0.54 (0.41, 0.71) **	0.86 (0.47, 0.93) *
P for trend	< 0.001	0.358	0.226	< 0.001	< 0.001	0.033
Daily dietary K intake						
Quartiles						
Q1 (low)	1 (Ref)	1 (Ref)	1 (Ref)	1 (Ref)	1 (Ref)	1 (Ref)
Q2	0.88 (0.69, 1.13)	0.90 (0.69, 1.16)	0.85 (0.65, 1.11)	0.84 (0.66, 1.07)	0.79 (0.61, 1.02)	0.84 (0.64, 1.10)
Q3	0.90 (0.70, 1.14)	0.94 (0.73, 1.22)	0.89 (0.67, 1.19)	0.84 (0.65, 1.07)	0.79 (0.61, 1.01)	0.90 (0.67, 1.21)
Q4 (high)	0.69 (0.53, 0.90) **	0.85 (0.64, 1.11)	0.74 (0.51, 1.05)	0.67 (0.52, 0.87) **	0.63 (0.48, 0.82) **	0.74 (0.52, 1.04)
P for trend	0.100	0.290	0.129	< 0.001	< 0.001	0.130

RA, rheumatoid arthritis; NHANES, National Health and Nutrition Examination Survey; *Q* quartile, *OR* odds ratio, *Mg* magnesium, *Cu* copper, *K* potassium

^*^ *p* < 0.05, ** *p* < 0.01

## Discussion

In this cross-sectional study, we investigated the association between dietary Mg, Cu and K with the risk of RA. Our results revealed that an increased intake of Mg, Cu and K were negatively associated with RA.

Different studies showed inconsistent conclusion on the association between Mg and the risk of RA. Arablou et al. demonstrated that Mg intake was negatively associated with the inflammatory factors of RA such as Prostaglandin E2 [15]. Another large NHANES study reported a U-shaped linking between Mg intake and RA in US women [22]. A dietary intake range of 181 ~ 464 mmol/day was found to remain the lowest prevalence of RA. It is reported that Iranian women took less Mg compared with dietary reference intake, although no significant statistical association between Mg intake and inflammatory markers were observed [23]. Cheng et al. conducted a mendelian randomization study and found that per-unit increase in blood Mg concentration were correlated to an 8.94-fold increased risk of RA [24]. Our findings contradict these results. According to previous knowledge, deficiency of Mg in blood cause inward flow of calcium ions which further results in increased stimulation of the N-methyl-d-aspartate (NMDA) receptor [25, 26]. This leads to the release of inflammatory medium such as Substance P and inflammatory cytokines including interleukin-6 and tumor necrosis factor [27–29]. However, more research is needed to fully understand the relationship between Mg and RA.

Several epidemiological studies and meta-analyses have extensively elucidated the link between Cu and RA [18, 30, 31]. However, to our knowledge, only a limited number of studies have focused on investigating the association between dietary Cu intake and RA. OM Silverio Amancio et al. found that juvenile RA characteristics do not significantly affect Cu intake, but they had intakes lower than the recommended levels [32]. This may indicate a greater deficit of dietary Cu due to the inflammatory process and the specific functions of Cu in inflammatory diseases [32]. Furthermore, another study suggested that dietary Cu was not significantly associated with the risk of RA, however, the utilization of Cu supplements showed an inverse relationship with the risk of RA [33]. The exact impact of Cu on RA remains uncertain, and further investigation is required to uncover the underlying mechanisms. Cu is an essential bioelement in numerous biochemical processes, serving as an integral component of several enzymes [34, 35]. Its involvement in anti-oxidative processes is well established, and it plays a vital role in the physiology of cells [36]. A study has reported a positive correlation between serum Cu levels and the overall disease activity in RA [37]. Additionally, Cu is a necessary mineral for bone development and maintenance, and is responsible for appropriate cartilage mineralization, elastin and collagen structure formation, bony trabeculation structure creation, and crosslinking of collagen and elastin [38–40]. Moreover, Cu also plays a significant role in immune response by supporting the activity and effectiveness of humoral and cellular immunity, including the production of IL-2 by activated lymphocytic cells [41, 42]. Consequently, it is conceivable that the connection between Cu consumption and RA is mediated by means of these biological pathways.

To the best of our knowledge, only a limited number of studies have investigated the potential correlations between dietary K intake and RA. Our study showed an inverse association between K and RA. Salivary K levels were found to be significantly reduced in the RA group [43]. An Indian study suggested that the dietary K content of RA patients was significantly lower than that of the healthy controls [44]. It is reported that RA patients experienced a statistically significant reduction in pain and inflammatory arthritis following the oral administration of K supplements [45]. Although the role of K intake in RA has not been fully elucidated, accumulating evidence demonstrated that adequate dietary K plays important roles in improving bone health [46–48].

One of the notable strengths of our study is the inclusion of a large and nationally representative sample of US adults. Furthermore, our findings indicate a significant inverse correlation between dietary Mg, Cu and K intakes, and RA in multivariate analyses, even after adjusting for several potential confounding factors. Additionally, we investigated the dose–response relationship between dietary metals and the risk of RA, which allowed us to further elucidate the relationship between metals intake and RA.

Some limitations of this study should be mentioned. First, the cross-sectional design of NHANES data limits our ability to establish causality between dietary trace element intake and RA development. While our study identified an inverse association between Mg, Cu and K and RA, it is possible that reverse causality may exist, where RA may affect metal intake through dietary changes or supplement use. Second, the NHANES data on dietary trace element intake relied on self-reported dietary recalls, which are subject to measurement error and recall bias. The dietary recalls may not accurately represent habitual dietary intake or account for day-to-day variation, leading to potential misclassification of dietary trace element intake. Third, our study only considered the association between dietary trace elements and RA, while other dietary factors and lifestyle factors, such as physical activity, may also play important roles in RA development. Therefore, our findings may be confounded by these factors, and future studies should consider these potential confounders in their analyses.

In conclusion, our results indicate that higher dietary Mg, Cu and K intakes may be inversely associated with the risk of RA in US adults. An inverse L-shaped association between dietary Cu and RA was found. Dietary Cu intake of 0.3–2.2 mg/day was associated with decreased relative odds of RA. Therefore, higher intake of Mg, Cu and K may be protective against RA. Due to the study's cross-sectional design, causal association regarding these findings could not be established. Further prospective cohort studies with larger sample sizes are needed to confirm our finding.

## Data Availability

Those datasets generated and analyzed in the current study can be found at NHANES website (https://www.cdc.gov/nchs/nhanes/index.htm). Accessed on 10 March 2023.
